# A clustering-based method for single-channel fetal heart rate monitoring

**DOI:** 10.1371/journal.pone.0199308

**Published:** 2018-06-22

**Authors:** Encarnación Castillo, Diego P. Morales, Antonio García, Luis Parrilla, Víctor U. Ruiz, José A. Álvarez-Bermejo

**Affiliations:** 1 Department of Electronics and Computer Technology, Campus Universitario Fuentenueva, University of Granada, Granada, Spain; 2 Department of Informatics, University of Almería, Almería, Spain; University of Minnesota, UNITED STATES

## Abstract

Non-invasive fetal electrocardiography (ECG) is based on the acquisition of signals from abdominal surface electrodes. The composite abdominal signal consists of the maternal electrocardiogram along with the fetal electrocardiogram and other electrical interferences. These recordings allow for the acquisition of valuable and reliable information that helps ensure fetal well-being during pregnancy. This paper introduces a procedure for fetal heart rate extraction from a single-channel abdominal ECG signal. The procedure is composed of three main stages: a method based on wavelet for signal denoising, a new clustering-based methodology for detecting fetal QRS complexes, and a final stage to correct false positives and false negatives. The novelty of the procedure thus relies on using clustering techniques to classify singularities from the abdominal ECG into three types: maternal QRS complexes, fetal QRS complexes, and noise. The amplitude and time distance of all the local maxima followed by a local minimum were selected as features for the clustering classification. A wide set of real abdominal ECG recordings from two different databases, providing a large range of different characteristics, was used to illustrate the efficiency of the proposed method. The accuracy achieved shows that the proposed technique exhibits a competitve performance when compared to other recent works in the literature and a better performance over threshold-based techniques.

## Introduction

The early detection of defects in the fetal heart is of paramount importance for the management of pregnancy and childbirth timing. In addition, it can help in the diagnosis of possible abnormalities in other organs. Among all fetal heart problems, heart rhythm abnormalities [1] occur in up to 2% of pregnancies and account for 10–20% of the referrals to fetal cardiologists [2]. Therefore, fetal heart rate (FHR) measurement is integral to fetal surveillance throughout pregnancy, as it is of significant clinical importance. Fetal electrocardiography [3, 4] can be used for the detection of the FHR before birth and thus makes it possible to administer faster medical or surgical interventions once the baby is born, if required. Noninvasive fetal electrocardiogram (FECG) monitoring methods [5, 6] measure the abdominal electrocardiogram (AECG) through skin electrodes on the expecting mother’s abdomen. This signal is composed of the FECG along with the maternal electrocardiogram (MECG). These measuring techniques have a greater prospect for the long-term monitoring of FHR and fetal well-being using signal processing techniques [7]. Among all the different methods, measuring the FHR from single-channel abdominal recordings [8–13] would be more convenient and have a lower cost, obtaining also a high accuracy. One of the most important difficulties for detecting FHR derives from the fact that the FECG signal is much weaker than other interfering biosignals (maternal cardiac signals, uterine contraction, fetal brain activity, or fetal and maternal myography) and movement artifacts [14]. Moreover, obtaining FECG signals from AECG signals is affected by large distortion from different types of interferences, especially the MECG signal, which is difficult to suppress without significantly degrading the FECG signal. Satisfactory results have been obtained in the removal of noise from AECG signals [15]. For example, Discrete Wavelet Transform (DWT) [16] structures can be used for the suppression of different types of noise, including direct current (DC) levels and wandering [14, 17, 18]. On the other hand, although techniques based on the elimination or separation of MECGs make FHR extraction possible, the results are not as good for obtaining more exhaustive information referring to the P waves and QRS complexes of the FECG signal. Considering this aspect, AECG signal processing can be oriented to extracting the same information as prior techniques, the FHR, but directly from the AECG signals, thus avoiding the processing required by MECG removal. Specifically, maternal and fetal QRS complexes can be localized by means of thresholds applied to the denoised AECG signal [19]. It is important to keep in mind that MECG and FECG amplitudes may be comparable in some cases. In addition, in abdominal signals, the fetal R-peaks often overlap with the maternal R-peaks. These facts make it difficult to develop a unique method that effectively detects the FHR from AECG signals. In this sense, the main drawback of threshold-based methods is the difficulty in determining the thresholds that would make the extensive use of AECG signals possible. The most problematic threshold is that related to amplitudes since, even with a normalization of the AECG signal, amplitude depends on several parameters, such as the pregnancy week, the position of the electrodes and the relative position of mother’s and fetus’ hearts. On the other hand, clustering techniques [20–22] have been used in many applications to identify sets of similar elements and to group them in clusters in such a way that elements in the same cluster are more similar to each other than to those in other clusters. Analyzing the graphical representation of each local maximum (R-peak candidate) followed by a local minimum (S-peak candidate) in AECG signals, clustering can be applied to differentiate between three different clusters: maternal RS-peaks, fetal RS-peaks and other waves (mainly noise). Thus, this paper proposes a new clustering-based technique for fetal QRS detection in AECG signals, avoiding the problems related to thresholds and enabling its automated application, leading to very high accuracy. Moreover, for FHR monitoring, the method uses only one abdominal electrode recording without separating the FECG from the AECG, so the fetal QRS complexes are directly extracted from the denoised AECG signal. The rest of the manuscript is organized as follows: the Methods Section first briefly describes the threshold-based method previously proposed [19] for fetal QRS detection and introduces some clustering fundamentals. The next subsection is devoted to the description of the clustering-based algorithm developed for the proposed FHR extraction method, detailing the selection of the parameters involved at each step. Results and discussion are presented in the following section, while the final section summarizes the conclusions of this work.

## Methods

### Threshold-based FQRS detection background

The main stages of the threshold-based, FQRS-detection method for FHR monitoring in [19] are wavelet-based preprocessing, maternal and fetal QRS detection, and false positive (FP) and false negative (FN) correction. The wavelet preprocessing method [14, 19], simultaneously eliminates baseline wandering (BW) and noise using only one wavelet decomposition and reconstruction structure. The application of the wavelet preprocessing approach consists of wavelet decomposition down to *L* levels [14], with the approximation coefficients at level *L* replaced by an all-zero vector. Additionally, for each level from *i* = 1 to *M* (with *M* < *L*), the appropriate threshold limit and rule [14] are applied to the detail coefficientsṪhe wavelet reconstruction based on the zeroing approximations of level *L*, the modified details of levels 1 to *M*, and the original details of levels *M* + 1 to *L* are computed to obtain the BW-corrected and denoised AECG signal.

The maternal and fetal QRS detection is based on the localization of maternal and fetal QRS complexes by means of thresholds [19]. For maternal QRS detection, the maximum of the preprocessed signal is identified as the R-peak of greatest amplitude in the maternal QRS complexes, which is used as referecence for identifying maternal QRS complexes as a certain percentage of this maximum. For fetal QRS detection, a search for local maxima (R-peak candidates) followed by a local minimum (S-peak candidate) between each two maternal QRS complexes is carried out. Time and amplitude thresholds are also applied to this search, resulting in RS-peak candidates. Time thresholds are related to extremely low FHR, while the amplitude threshold is established within a range related to the maximum of MQRS complexes after BW and noise suppression. Candidate RS-peaks meeting these two thresholds will be stored as FQRS candidates.

The FHR monitoring finally includes a method for the detection of false negatives (FN, a non-detected FQRS complex) and false positives (FP, a false-detected FQRS complex) [19] [23]. For each FQRS candidate, several RR time distances are defined and checked, and FPs and FNs are detected according to heart rate limits [19].

Some of the steps required by the method proposed in [19] are based on the selection or calculation of thresholds. These thresholds themselves constitute a major disadvantage for these methods, as it is difficult to ensure that any selected or calculated threshold is the most appropriate for the signal under study. Thus, amplitude thresholds have to be either selected according to certain known parameters (such as pregnancy week, position of the fetus or electrode positions) or manually adapted according to the characteristics of each signal. This is usually the case when working with different public databases, as it has been found that it is difficult to establish a fixed relationship between different thresholds and signal parameters, even when the data are normalized, since thresholds also depend on unknown parameters (from the noise affecting the abdominal signal to biological characteristics). Moreover, fetal QRS complexes may be comparable in amplitude to maternal QRSs. This all is a significant barrier to automating threshold-based methods for real-time FHR monitoring. Therefore, new alternatives were evaluated for maternal and fetal QRS extraction in order to avoid these difficulties, with clustering techniques [21] being one of the best candidates. The new method uses a clustering procedure to classify certain information obtained from the denoised abdominal ECG signal into three clusters: maternal RS-peaks, fetal RS-peaks and other waves.

### Fetal ECG datasets

PhysioNet [24] offers free and public web access to large collections of recorded physiologic signals (PhysioBank) and the included databases are made available under the ODC Public Domain Dedication and License v1.0 [25]. Concretely, two of these databases provided by Physionet [24] have been used for this work:

- *Abdominal and Direct Fetal Electrocardiogram Database* [24] [26]: this database contains multichannel FECG recordings obtained from 5 different women in labor. Each recording comprises four 5-minute differential signals acquired from maternal abdomen and the reference direct fetal electrocardiogram registered from the fetal head. The recordings are sampled at 1 ksps with 16-bit resolution, and the signal bandwidth is 1-150 Hz. Moreover, the database includes a set of reference annotations indicating the fetal R-wave locations.
- *Challenge 2013 Training Set A* [24] [27]: these data consist of one-minute fetal ECG recordings, sampled at 1 ksps, each one including four noninvasive abdominal signals as well as the reference annotations marking fetal R-waves locations.

### Clustering fundamentals

Clustering [21, 22] is one of the most important unsupervised learning techniques for the classification of objects into clusters. A cluster is therefore a collection of similar objects that are dissimilar to the objects belonging to other clusters. One possible measure of relative dissimilarity is the distance *D*(*x*_*i*_, *x*_*j*_) between two objects *x*_*i*_ and *x*_*j*_. Therefore, given a clustering algorithm and according to the distance function, a group *X* of *n* objects, *X* = [*x*_0_, …, *x*_*j*_, …, *x*_*n*_], where each observation is a *d*-dimensional real vector *x*_*j*_ = (*x*_*j*1_, …, *x*_*jd*_)^*T*^ ∈ *R*^*d*^, is divided into *k* clusters, *C* = [*c*_1_, …, *c*_*k*_], where $\cup_{x=1}^{k}c_{i}=X$, and ∀*i* ≠ *j*, *c*_*i*_ ∩ *c*_*j*_ = ⌀. A distance function over a data group *X* is defined to satisfy the reflexivity, symmetry, positivity and Minkowski’s inequality conditions [28]. The Minkowski distance comprises a family of metrics defined traditionally to measure distances. The Manhattan, Euclidean, and Chebyshev distances [29] are special cases of the Minkowski distance when *p* = 1, *p* = 2 and *p* → ∞, respectively.

A distinction among different types of clusterings is whether the set of clusters is nested or unnested. Thus, clustering may be classified as hierarchical clustering [30] or partitional clustering [31]. Partitional clustering is popular in various research fields [22] due to its capability to cluster large datasets, and this is the clustering type used in the present work. It divides the set of data objects into non-overlapping clusters, given certain criteria, with each cluster represented by its centroid. The *k-means* algorithm [32] is the most fundamental partitional clustering concept, the optimization criterion of which is the minimization of the Euclidean distance between elements and cluster. Inspired by *k-means*, several gradient algorithms for partitional clustering have been developed by researchers, for example, *k-means++*, which we selected as the clustering algorithm to classify the information extracted from AECG signals.

### Clustering-based procedure for FHR extraction

**Fig 1:**
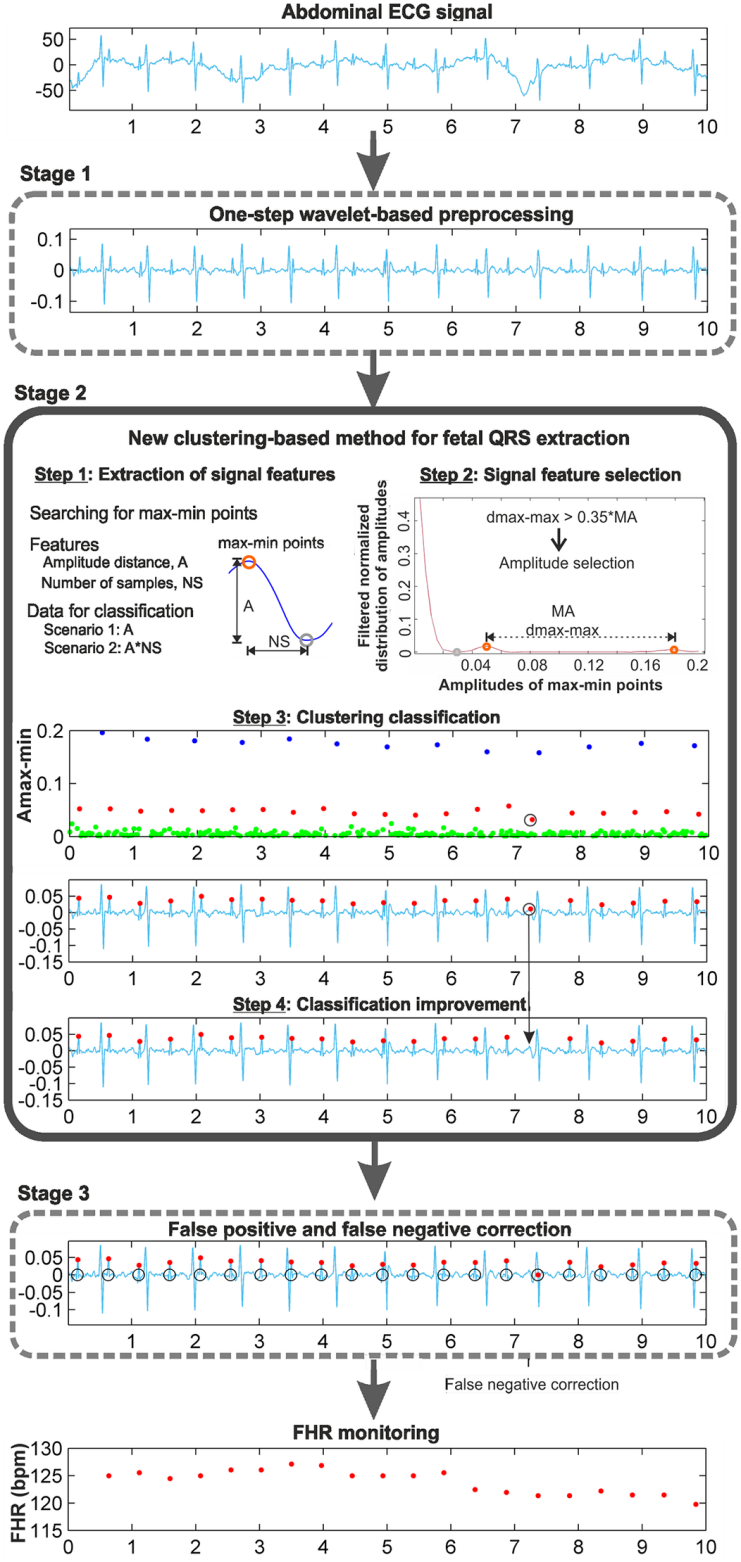
Clustering-based procedure for FHR monitoring.

A schematic of the new clustering-based procedure is shown in Fig 1. The procedure is mainly composed of three stages, corresponding to each frame in the figure: two of the previously developed stages, the wavelet-based preprocessing stage, the FP and FN correction stage, and the new clustering-based method for fetal QRS extraction. This new stage is fed with the denoised AECG signal, and after extracting the fetal QRS complexes, it sends them to the FP and FN correction stage. The proof of concept has been implemented using MATLAB and validated using real AECG recordings from the fetal ECG datasets mentioned above. The different steps of this clustering stage are illustrated in Fig 1 making use of the r04 Ab-2 recording of the *Abdominal and Direct Fetal Electrocardiogram Database*. These steps are described in detail below.

#### Step 1: Extraction of signal features

**Fig 2:**
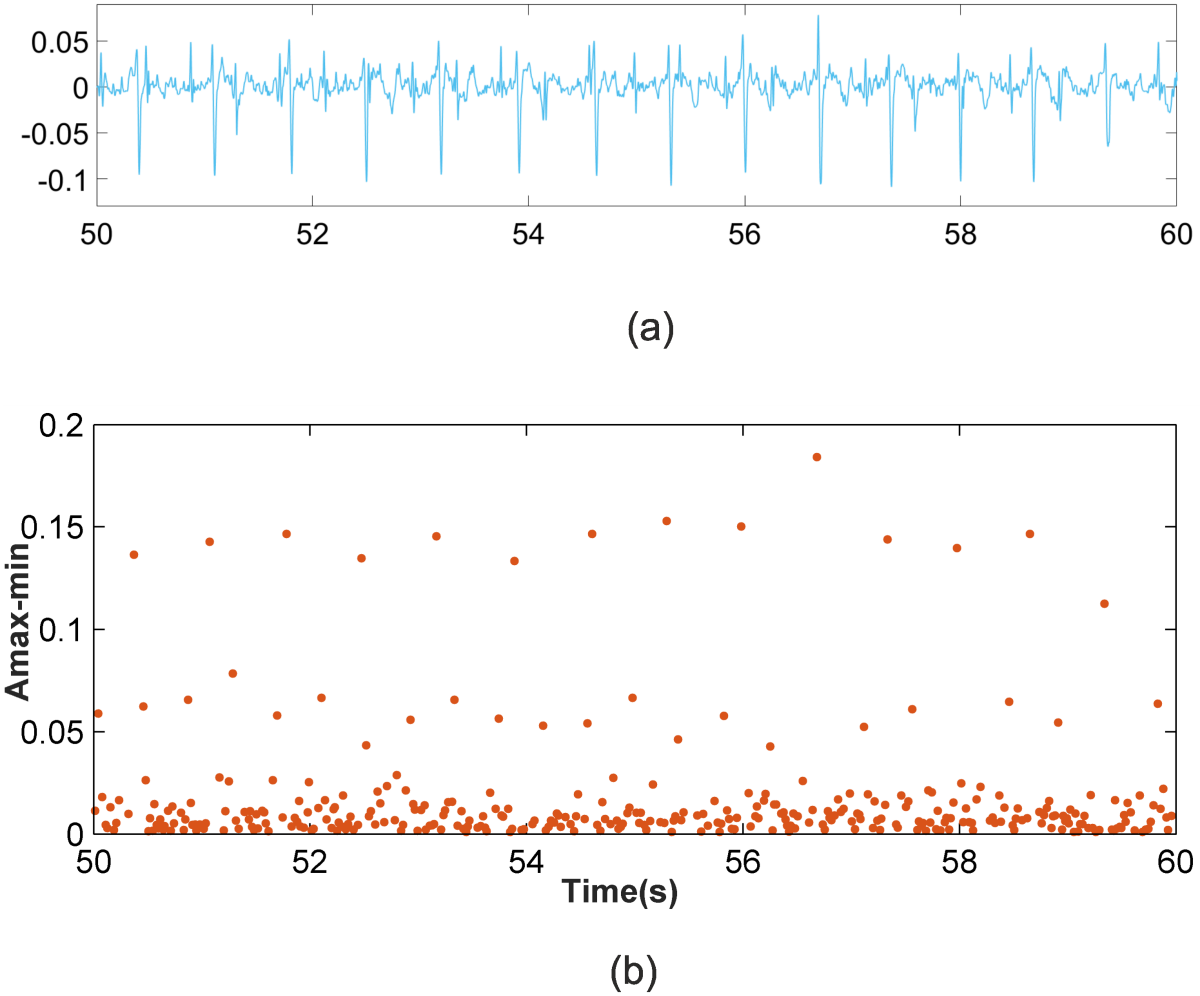
Scenario 1 example. (a) r08 Ab-1 preprocessed signal (b) Amplitude distance between the detected maximum followed by a minimum.

**Fig 3:**
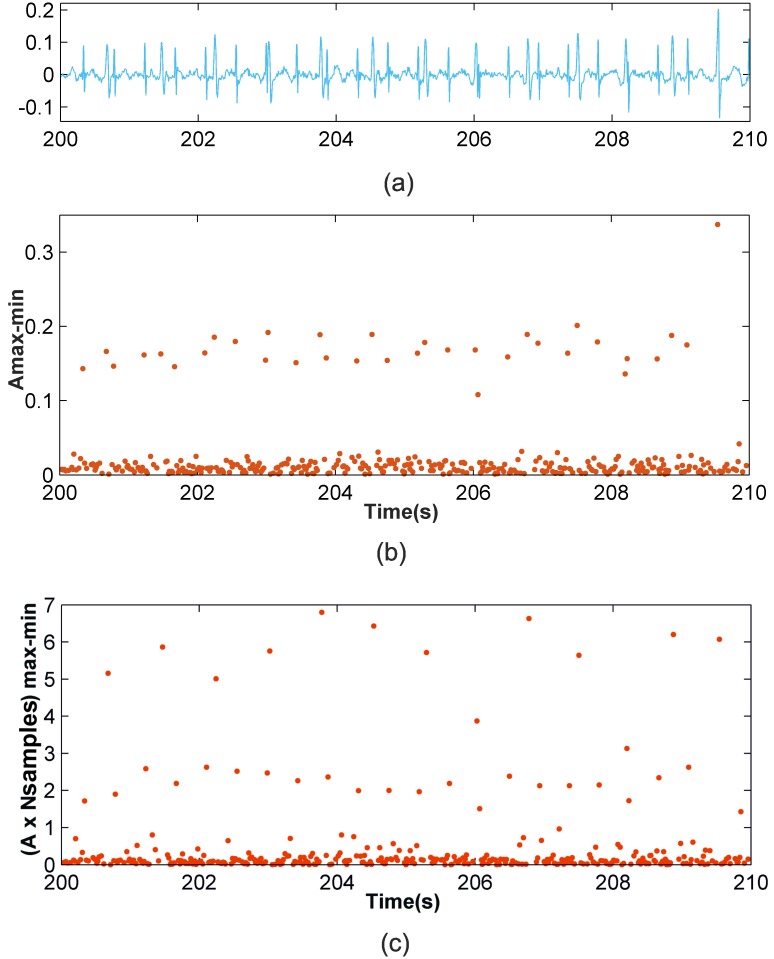
Example of Scenario 2. (a) r08 Ab-4 preprocessed signal (b) Amplitude distance of the detected maximum followed by a minimum (c) Amplitude distance multiplied by the number of samples of the detected maximum followed by a minimum.

Distinctive features of the denoised AECG signal must be selected for the application of the clustering algorithm. This selection is crucial for the effectiveness of the clustering application. For this task, it is necessary to analyze the signal and to identify the points or zones of this signal that contain the information of interest. Our objective is to extract the FHR, which requires localizing the fetal QRS complexes. Analyzing the denoised AECG signals, it can be observed that for the identification of fetal QRS complexes, the most noticeable characteristics are the RS-peaks. From the QRS morphology, it can be observed that the RS-peak is a local maximum followed by a local minimum, as shown in Fig 1, at the extraction of signal features step. In this way, a search for local maxima followed by a local minimum in the preprocessed AECG signal is made, resulting in the max-min points. Hence, the objective of our clustering classification consists of using certain features of these max-min points to classify them adequately into three clusters. Analysing the 5-minute recordings of the training database and their main characteristics, the amplitude and time distances of these max-min points have been selected as candidate features, and two different scenarios have been considered:

- Scenario 1: This scenario considers signal windows where the maternal RS amplitudes are larger than the fetal RS amplitudes. This scenario allows for the use of the amplitude distance between the max-min points as the data to be classified. Fig 2 shows a representation of the amplitude distance between the max-min points located in a set of 10,000 samples from the r08 Ab-1 denoised recording from the *Abdominal and Direct Fetal Electrocardiogram Database* [24], [26]. In Fig 2b the *X*-axis corresponds to the time where each maximum of the max-min points occurs, and the *Y*-axis corresponds to the amplitude distance. From this figure, three different horizontal zones can be clearly distinguished, related to the maternal RS-peaks, fetal RS-peaks and other waves. As such, for this case, the amplitude distance between the max-min points is a good option for classification.
- Scenario 2: This other scenario considers signal windows where fetal RS amplitudes are similar to maternal RS amplitudes so maternal and fetal RS-peaks would likely be classified in the same cluster. It was observed from the AECG signals that the maternal RS time distance is sufficiently larger than the fetal RS time distance to allow for visual differentiation between the fetal and maternal RS-peaks. Accordingly, the amplitude distance would allow for the differentiation of maternal and fetal RS-peaks from noise and other wave information. Meanwhile, the RS time distance, or the RS number of samples, is an important parameter that allows us to distinguish between fetal and maternal QRS complexes. After carrying out a detailed study, we found that very satisfactory classification results are achieved using the amplitude multiplied by the number of samples of max-min points as the data to be classified. Fig 3 shows similar information to that shown in Fig 2b but for the denoised r08 Ab-4 signal. From Fig 3b, it can be observed that using the amplitude feature, only two horizontal zones can be visually distinguished. The zone with larger amplitudes corresponds to mixed maternal and fetal RS-peaks, and the other is related to other waves and noise. This is due to this signal presenting similar max-min point amplitudes for fetal and maternal QRS complexes. Thus, it is necessary to also use another feature to correctly classify the data into three clusters. Fig 3c displays the obtained graphic with the amplitude distance multiplied by the number of samples of the max-min points on the *Y*-axis. From this figure, it is possible to differentiate the three horizontal zones of interest.

#### Step 2: Signal feature selection

**Fig 4:**
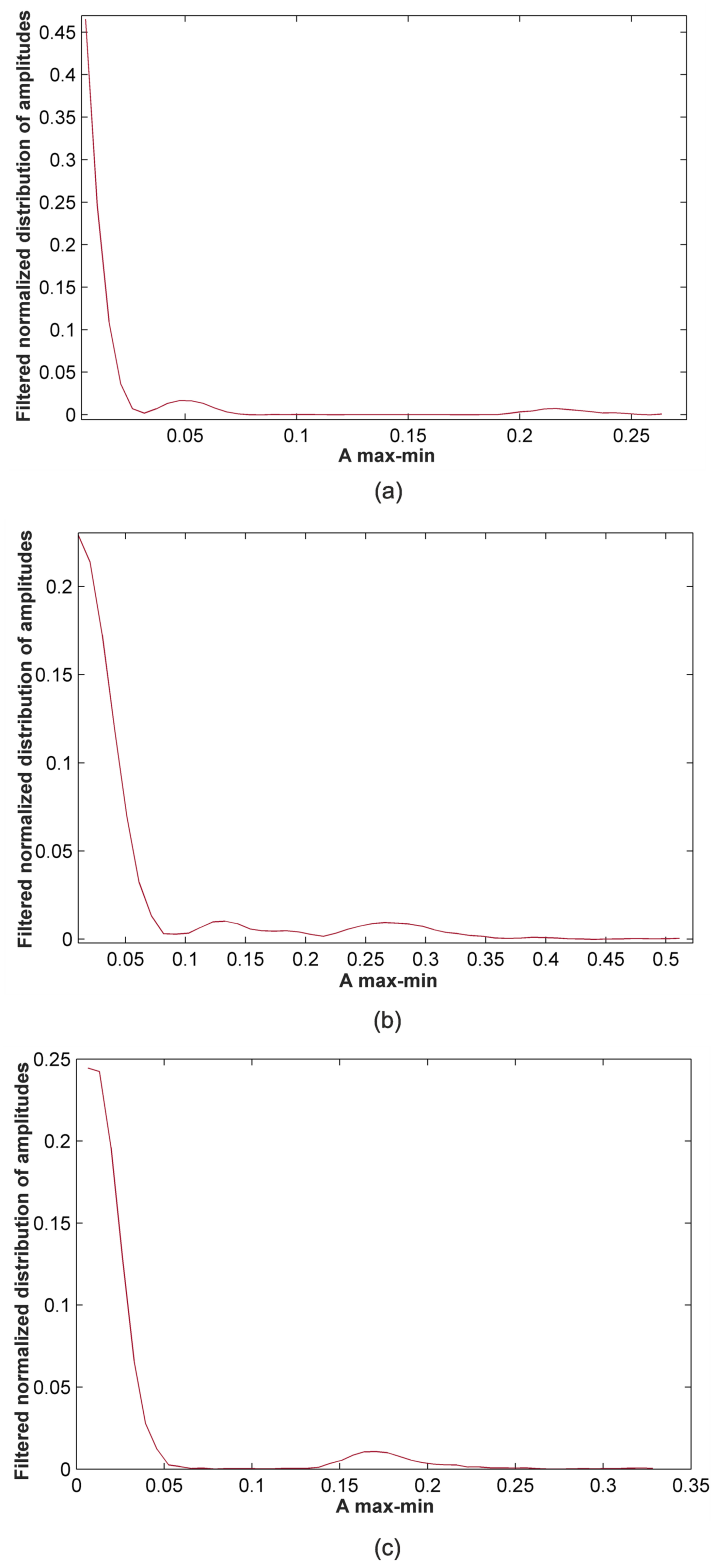
Example of feature selection using the filtered normalized distribution of amplitudes. (a) Case 1: selection of amplitude (50,000-sample window of r07 Ab-4 denoised recording) (b) Case 2: selection of amplitude multiplied by the number of samples (50,000-sample window of r01 Ab-4 denoised recording) (c) Case 3: selection of amplitude multiplied by the number samples (50,000-sample window of r08 Ab-4 denoised recording).

In order to automatically detect which of the scenarios detailed above corresponds to the signal or, more specifically, to the data window to be processed, we have used a procedure based on the distribution of the amplitudes of the detected max-min points:

- Calculate the maximum value of the amplitudes of the max-min points, called MA.
- Divide the range from 0 to MA into 50 intervals, which was found to be an optimum partitioning over a wide range of training sets.
- Calculate the number of amplitudes that are within each interval, *i.e.*, calculate the distribution of amplitudes.
- Normalize the distribution of amplitudes by dividing them by the total number of the max-min points.
- Apply a smoothing filter to the normalized distribution of amplitudes. Fig 4a shows the filtered normalized distribution of amplitudes obtained for a 50,000-sample window of the denoised r01 Ab-1 AECG signal.
- Detect the local maxima and local minima of the filtered normalized distribution of amplitudes. In general, from these points, we have to identify three different cases:
  - Case 1: Detection of two local maxima from the first local minimum. Each of these maxima provides information about the amplitude zones corresponding to fetal RS-peaks and maternal RS-peaks. After the data training, it was concluded that if the distance between these maxima, called dmax-max, is 35% greater than the MA value, we are under Scenario 1. In this case, amplitudes are the selected data to be classified. Fig 4a illustrates this case. As can be observed in this Figure, the identified maxima are separated by more than the 35% of the MA.
  - Case 2: Detection of two local maxima from the first local minimum, the distance between which is 35% less than the MA value. This situation can be related to Scenario 2, and thus, amplitudes multiplied by the number of samples of the max-min points are data to be classified. An example of this case is presented in Fig 4b.
  - Case 3: Detection of a single local maximum from the first local minimum. This situation generally corresponds to Scenario 2, as amplitudes for fetal and maternal RS-peaks are so similar that they are located in only one zone, with the located maximum being representative of this zone. For this case, amplitudes multiplied by the number of samples of the max-min points are the selected data to be classified. Fig 4c displays an example of this case.
  For all other cases, the amplitude of the max-min points was selected as the data to be classified.

#### Step 3: Clustering classification

After having studied the features of the signal and having defined the data to be classified, it was necessary to choose the clustering algorithm to be used and its parameters. Taking into account the results obtained from a preliminary study [33], we have focused our interests on *k-medoids*++. We have used the *kmedoids* function from the Statistics and Machine Learning Toolbox in MATLAB. The main input data of this function are:

- Data to be classified: these data are selected from the previous step.
- Number of clusters: this is equal to 3, as the data have to be classified into three clusters: maternal RS-peaks, fetal RS-peaks and other waves and noise.
- Distance measure: the dissimilarity between the data have to be measured using distance functions, which were introduced in the previous subsection. Taking into account that the data to be classified are unidimensional, for *d* = 1 Minkowski, Manhattan, Euclidean and Chebyshev distances are all defined as *D*(*x*_*i*_, *x*_*j*_) = ∣*x*_*i*_ − *x*_*j*_∣. In clustering algorithms, it is common to use an alternative measure of the Euclidean distance, the squared Euclidean distance, *D*^2^(*x*_*i*_, *x*_*j*_) = (*x*_*i*_ − *x*_*j*_)^2^, which places progressively greater weight on objects that are farther apart. This squared Euclidean distance is not a metric but is frequently used in optimization problems in which distances only have to be compared. Thus, the squared Euclidean distance was applied for our classification.
- Number of times to repeat the clustering using new initial cluster medoid positions: this value was experimentally fixed to 20 using training sets.
- Method for choosing the initial cluster medoid positions: as the *k-medoids*++ clustering algorithm was chosen, this input was selected as ‘plus’ (++).

The *kmedoids* function returns a vector containing cluster indices that were used to separate these data into three clusters. The cluster corresponding to the fetal RS-peaks was identified as that with a median value situated between the median values of the other two clusters.

#### Step 4: Classification improvement

**Fig 5:**
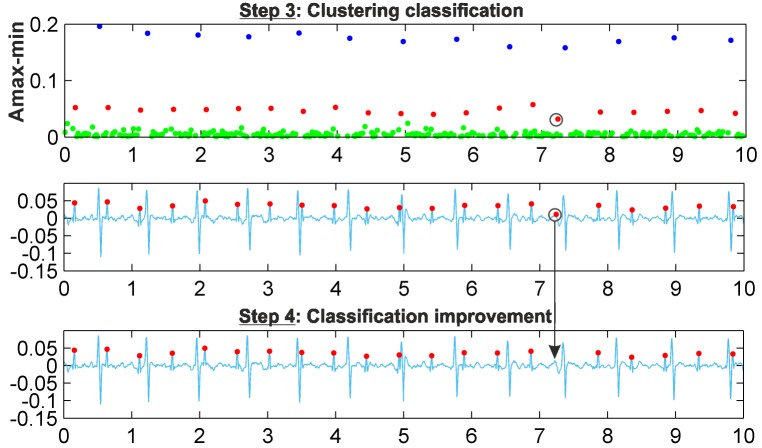
Clustering improvement example.

**Fig 6:**
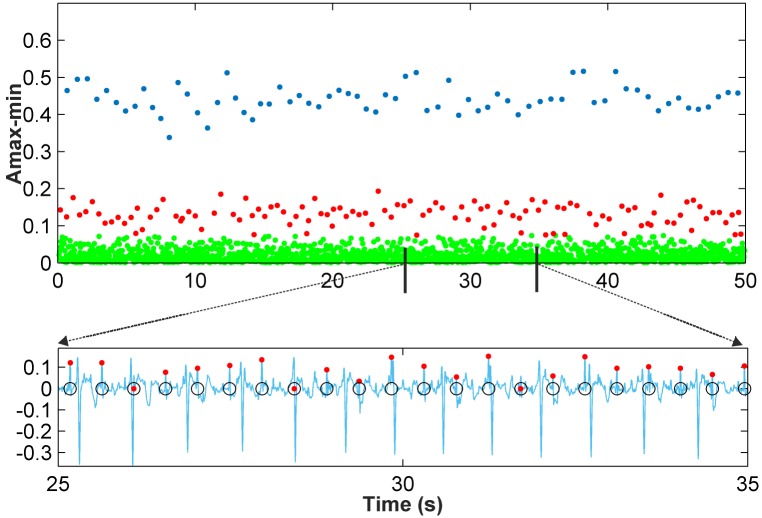
Clustering classification for r01 Ab-1 recording.

**Fig 7:**
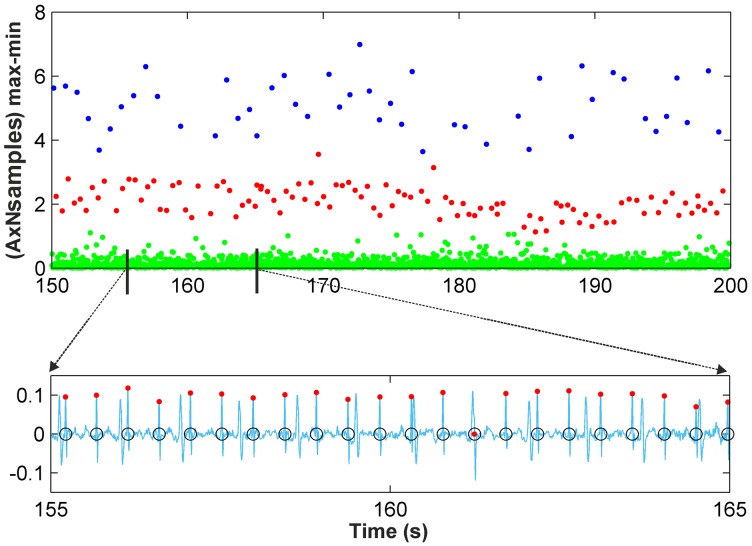
Clustering classification for r08 Ab-1 recording.

A final stage to improve classification results is carried out, mainly consisting of the imposition of some limits on the amplitude and time distance of the data classified as fetal RS-peaks. These limits are calculated using the local points detected from the filtered normalized distribution of amplitudes. Thus, the first graphic in Fig 5 shows the max-min points of each resulting cluster with different colors, with blue points corresponding to maternal RS-peaks and red points to fetal RS-peaks, while green points are related to other waves. The second graphic displays the denoised signal and the detected fetal RS-peaks (red points). The marked max-min point (black circle) was classified as a fetal RS-peak, but it is not fetal, as it corresponds to other waves. As can be observed in the graphic of the classification improvement step, this max-min point is eliminated from the fetal cluster, thus showing the effectiveness of this improvement step. Different recordings were used as a training set to establish the optimal value of the number of samples in this data window for the clustering classification. This study indicated that in general, from 10,000- to 60,000-sample windows, there was no noticeable difference between the effectiveness of the classification results. From this value range, we have selected 50,000-sample windows. It should be noted that Fig 5 uses a 10.000-sample window to clearly show the detected fetal R-peak. Fig 6 shows the results of *k-medoids*++ classification for a 50,000-sample window and the denoised signal, including the max-min points classified as fetal RS-peaks (red dots) and the annotations of the database (black circles). For this signal, the amplitude of the max-min points was the automatically selected feature for the clustering classification. Fig 7 shows similar results to those in Fig 6, but for this signal, as fetal and maternal RS amplitudes are very similar, the amplitude multiplied by the number of samples of the max-min points was the automatically selected feature for the clustering classification.

Finally, the data identified as fetal RS-peaks are ready to be processed by the FP and FN correction stage [19].

## Results and discussions

The parameters selected to assess the performance of this clustering-based proposal are sensitivity, *Se*, positive diagnostic value, *PPV*, accuracy, *Acc* [23], and *F*_1_-measure [34]:

$$Se=\frac{TD}{TD+FN}$$ (1)

$$PPV=\frac{TD}{TD+FP}$$ (2)

$$Acc=\frac{TD}{TD+FN+FP}$$ (3)

$$F_{1}=2\cdot \frac{PPV\cdot Se}{PPV+Se}=\frac{2\cdot TP}{2\cdot TP+FN+FP}$$ (4)

where TD means true detected fetal QRS complexes, and FP and FN are false negatives and false positives, respectively, that are found after the FP and FN correction stage. To decide when an obtained FQRS complex corresponds to a true FQRS complex, a fixed criterion is set. Thus, we discard all candidates differing by more than 50 ms from the reference annotation and mark them as FPs. Thus, *Se*, *PPV* and *Acc* are calculated from the fetal QRS complexes extracted by the proposed method having the annotations by the specialists in the databases as references.

The *Abdominal and Direct Fetal Electrocardiogram Database*, which was used to train the clustering-based stage, was also used to train the complete method, since it includes R-wave markers that allow the calculation of accuracy parameters. When reviewing other works in the field [8–10, 13], authors neglect recordings where the FECG is hardly detectable or make a selection of some recordings and channels for their studies. For this work, a medical specialist determined the clinical interest of the AECG signals for our study. The cardiology specialist thus excluded from the study some of the AECG abdominal signals where fetal heart beats were not detectable, and signals that were affected by severe artifacts, and even saturation, or were made of no clinical interest by severe noise. These excluded signals were r04 Ab-1, r07 Ab-1 and r10 Ab-3. At the same time, the rest of the database signals, all included in the study, were classified in two groups: first, those with no evident problems or artifacts (group 1), opposed to a second group of recordings presenting segments with big or repetitive artifacts or segments where it is extremely difficult to differentiate maternal from fetal PQRS complexes or to detect fetal complexes (group 2). On the other hand, the selected parameters for the wavelet preprocessing stage were wavelet function db6, *M* = 3, universal threshold, single rescaling and soft thresholding, with *L* = 7 [14, 19]. Table 1 presents the results obtained for the selected recordings of this dataset, classified in the two groups discussed above. Additionally, Table 1 also includes three different statistical summarizations of results: total data for all the analysed recordings, data for only recordings in group 1 (not affected by severe artifacts), and data for the best performing channel in each recording (shown in bold characters in Table 1), since some authors select for each recording a representative channel for the statistical summarizations of results [9, 10, 13]. Thus, selecting the best performing channel in each recording as representative data, *Se*, *PPV*, *Acc* and *F*_1_ are 98.40%, 98.86%, 97.30% and 98.63%, respectively, when the proposed clustering-based classification is applied. These performance data comprise a total of 3,182 FQRSs, of which 51 were not detected (FN 1.60%) and 36 were falsely detected as FQRS (FP 1.13%). When all the signals in Group 1 in Table 1 are considered, with a total of 6,990 FQRSs resulting in 175 FNs (2.50%) and 98 FPs (1.40%), *Se*, *PPV*, *Acc* and *F*_1_ are 97.50%, 98.58%, 96.15% and 98.04%, respectively. These results validate the proposed procedure and its capabilities.

**Table 1:** Evaluation results using *Abdominal and Direct Fetal ECG Database* 5-minute recordings as training data (data for the best performing channel in each recording are shown in bold characters).

Recording	T FQRS	TP	FN	FP	*Se*(%)	*PPV*(%)	*Acc*(%)	*F*_1_(%)
**Group 1: signals selected by a medical specialist**								
r01 Ab-1	643	631	12	8	98.13	98.75	96.93	98.44
**r01 Ab-4**	**643**	**637**	**6**	**2**	**99.07**	**99.69**	**98.76**	**99.38**
**r04 Ab-2**	**631**	**614**	**17**	**13**	**97.31**	**97.93**	**95.34**	**97.62**
r04 Ab-4	631	609	22	13	96.51	97.91	94.57	97.21
r07 Ab-2	626	603	23	12	96.33	98.05	94.51	97.18
r07 Ab-3	626	607	19	8	96.96	98.70	95.74	97.82
**r07 Ab-4**	**626**	**619**	**7**	**6**	**98.88**	**99.04**	**97.94**	**98.96**
r08 Ab-1	650	616	34	9	94.77	98.56	93.47	96.63
**r08 Ab-4**	**650**	**644**	**6**	**6**	**99.08**	**99.08**	**98.17**	**99.08**
r10 Ab-1	632	618	14	12	97.78	98.10	95.96	97.94
**r10 Ab-2**	**632**	**617**	**15**	**9**	**97.63**	**98.56**	**96.26**	**98.09**
**Group 2: signals afected by artifacts and noise**								
r01 Ab-2^a^	643	478	165	77	74,34	86,13	66.39	79,80
r01 Ab-3^b^	643	608	35	28	94.56	95.60	90.61	95.07
r04 Ab-3^b^	631	548	83	31	86.85	94.65	82.78	90.58
r08 Ab-2^a^^,^^c^	650	463	187	91	71.23	83.57	62.48	76.91
r08 Ab-3^c^	650	512	138	41	78.77	92.59	74,10	85.12
r10 Ab-4^b^	632	576	56	47	91.14	92.46	84.83	91.79
**Total**	10,839	10,000	839	413	92.26	96.03	88.87	94.11
**Group 1**	6,990	6,815	175	98	97.50	98.58	96.15	98.04
**Best channel**	3,182	3,131	51	36	98.40	98.86	97.30	98.63

^*a*^ Segments with undifferentiated maternal and fetal PQRS complexes

^*b*^ Segments with artifacts

^*c*^ Segments with saturation

**Fig 8:**
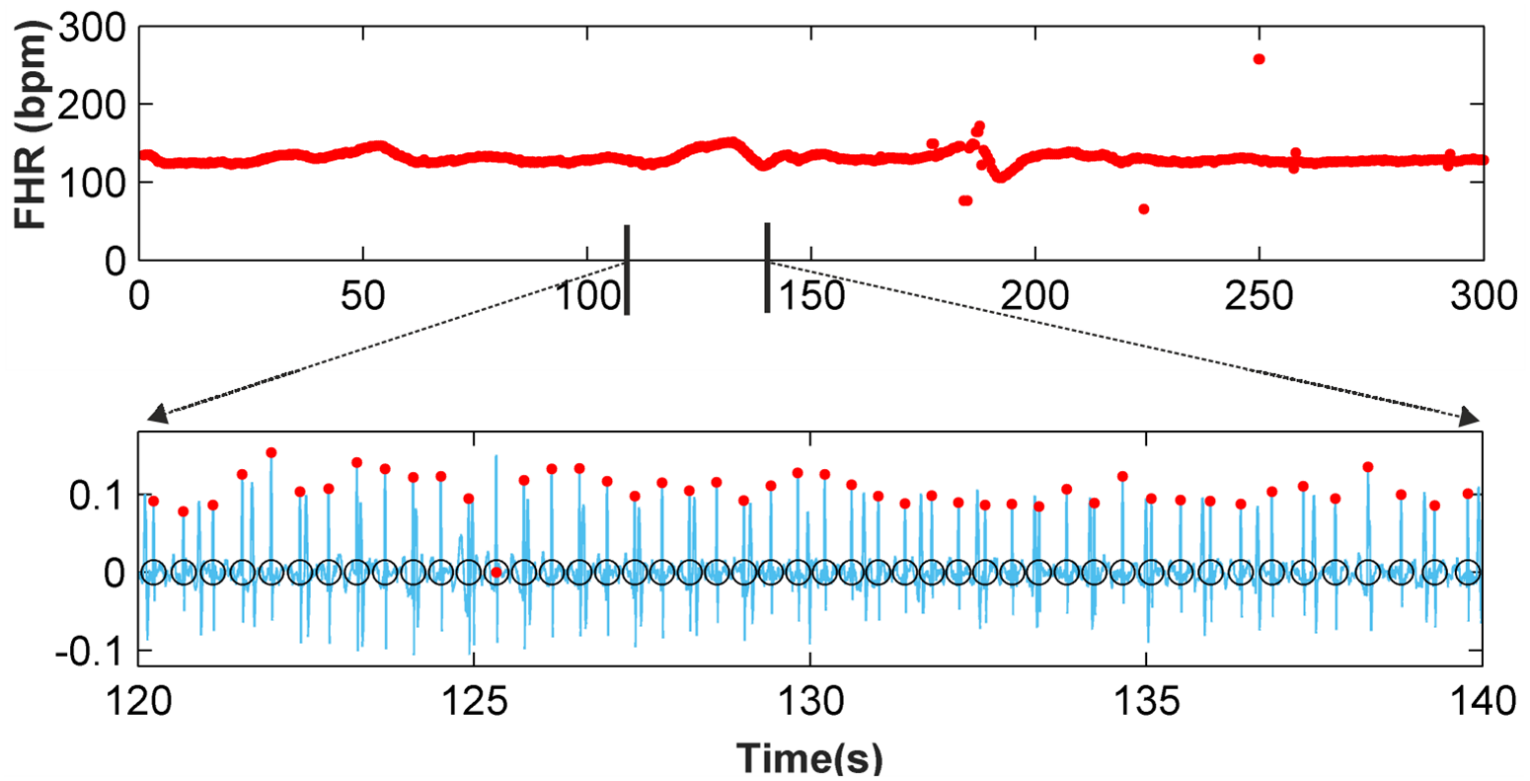
Five-minute FHR monitoring for r08 Ab-4 AECG recording and 20-second fetal QRS complex detection.

Fig 8 shows an example of FHR monitoring of the r08 Ab-4 AECG signal. In the first subplot, it can be seen that the proposed method is able to detect changes in the FHR with high accuracy. The second subplot shows 20 seconds of the denoised signal, including the detected FQRS complexes (red dots) and fetal annotations (black circles).

The *Challenge 2013 Training Set A* was used as testing data. As for the trainig database, a cardiology specialist selected a set of recordings from this testing database. All parameters for the wavelet preprocessing stage were the same as those detailed for the *Abdominal and Direct Fetal Electrocardiogram Database*. The results obtained from the 64 recordings selected by the specialist are summarized in Table 2. Moreover, Table 2 also includes two different statistical summarizations of results: data for all the analysed recordings and data for the best performing channel in each recording (shown again in bold characters in Table 2). The *Se*, *PPV*, *Acc* and *F*_1_ for the best performing channels are 97.93%, 99.11%, 97.08% and 98.52%, respectively. It is important to note that the processed signals include a wide range of different characteristics, and some include artifacts and very noisy fragments that were not eliminated from the results in Table 2. Even so, the obtained accuracy corroborates the efficiency of the method.

**Table 2:** Evaluation results using *Challenge 2013 Training Set A* 1-minute recordings as testing data (data for the best performing channel in each recording are shown in bold characters).

Recording	T FQRS	TP	FN	FP	*Se*(%)	*PPV*(%)	*Acc*(%)	*F*_1_(%)
**a03 Ab-1**	**127**	**127**	**0**	**0**	**100**	**100**	**100**	**100**
a03 Ab-2	127	124	3	5	97.64	96.12	93.94	96.88
a03 Ab-4	127	125	2	4	98.43	96.90	95.42	97.66
a04 Ab-1	128	126	2	3	98.44	97.67	96.18	98.05
a04 Ab-3	128	124	4	4	96.88	96.88	93.94	96.88
**a04 Ab-4**	**128**	**128**	**0**	**2**	**100**	**98.46**	**98.46**	**99.22**
a05 Ab-1	128	124	4	1	96.88	99.20	96.12	98.02
a05 Ab-3	128	124	4	2	96.88	98.41	95.38	97.64
**a05 Ab-4**	**128**	**128**	**0**	**0**	**100**	**100**	**100**	**100**
**a08 Ab-3**	**127**	**127**	**0**	**0**	**100**	**100**	**100**	**100**
a08 Ab-4	127	126	1	1	99.21	99.21	98.44	99.21
**a12 Ab-1**	**137**	**137**	**0**	**1**	**100**	**99.28**	**99.28**	**99.64**
a12 Ab-2	137	137	0	1	100	99.28	99.28	99.64
**a13 Ab-2**	**125**	**125**	**0**	**0**	**100**	**100**	**100**	**100**
a13 Ab-3	125	120	5	1	96.00	99.17	95.24	97.56
a13 Ab-4	125	122	3	2	97.60	98.39	96.06	97.99
**a14 Ab-1**	**122**	**119**	**3**	**2**	**97.54**	**98.35**	**95.97**	**97.94**
**a20 Ab-2**	**130**	**130**	**0**	**0**	**100**	**100**	**100**	**100**
a20 Ab-3	130	123	7	1	94.62	99.19	93.89	96.85
**a22 Ab-1**	**125**	**125**	**0**	**0**	**100**	**100**	**100**	**100**
a22 Ab-4	125	125	0	0	100	100	100	100
**a23 Ab-2**	**125**	**123**	**2**	**1**	**98.40**	**99.19**	**97.62**	**98.80**
a23 Ab-3	125	121	4	2	96.80	98.37	95.28	97.58
a23 Ab-4	125	123	2	2	98.40	98.40	96.85	98.40
a24 Ab-2	122	120	2	2	98.36	98.36	96.77	98.36
**a24 Ab-3**	**122**	**122**	**0**	**0**	**100**	**100**	**100**	**100**
a24 Ab-4	122	122	0	0	100	100	100	100
**a25 Ab-2**	**124**	**124**	**0**	**0**	**100**	**100**	**100**	**100**
a28 Ab-1	166	158	8	1	95.18	99.37	94.61	97.23
**a28 Ab-2**	**166**	**160**	**6**	**0**	**96.39**	**100**	**96.39**	**98.16**
a28 Ab-3	166	159	7	3	95.78	98.15	94.08	96.95
a35 Ab-1	162	158	4	3	97.53	98.14	95.76	97.83
a35 Ab-2	162	158	4	3	97.53	98.14	95.76	97.83
a35 Ab-3	162	158	4	3	97.53	98.14	95.76	97.83
**a35 Ab-4**	**162**	**161**	**1**	**4**	**99.38**	**97.58**	**96.99**	**98.47**
a36 Ab-1	167	164	3	0	98.20	100	98.20	99.09
a36 Ab-2	167	166	1	0	99.39	100	99.40	99.70
**a36 Ab-3**	**167**	**167**	**0**	**0**	**100**	**100**	**100**	**100**
a36 Ab-4	167	163	4	1	97.60	99.39	97.02	98.49
**a44 Ab-1**	**162**	**162**	**0**	**0**	**100**	**100**	**100**	**100**
a44 Ab-2	162	157	5	1	96.91	99.37	96.32	98.13
a44 Ab-3	162	157	5	3	96.91	98.13	95.15	97.52
a44 Ab-4	162	160	2	1	98.77	99.38	98.16	99.07
a49 Ab-1	147	145	2	1	98.64	99.32	97.97	98.98
**a49 Ab-2**	**147**	**147**	**0**	**0**	**100**	**100**	**100**	**100**
a49 Ab-3	147	147	0	0	100	100	100	100
**a55 Ab-2**	**142**	**135**	**7**	**3**	**95.07**	**97.83**	**93.10**	**96.43**
a55 Ab-3	142	128	14	6	90.14	95.52	86.49	92.75
a61 Ab-2	139	133	6	7	95.68	95.00	91.10	95.34
**a61 Ab-4**	**139**	**132**	**7**	**0**	**94.96**	**100**	**94.96**	**97.42**
a62 Ab-2	143	141	2	1	98.60	99.30	97.92	98.95
**a62 Ab-3**	**143**	**142**	**1**	**1**	**99.30**	**99.30**	**98.61**	**99.30**
a62 Ab-4	143	138	5	3	96.50	97.87	94.52	97.18
a65 Ab-2	143	130	13	8	90.91	94.20	86.09	92.53
**a65 Ab-4**	**143**	**138**	**5**	**1**	**96.50**	**99.28**	**95.83**	**97.87**
**a66 Ab-3**	**129**	**119**	**10**	**4**	**92.25**	**96.75**	**89.47**	**94.44**
**a67 Ab-4**	**153**	**135**	**18**	**7**	**88.24**	**95.07**	**84.38**	**91.53**
**a69 Ab-1**	**148**	**136**	**12**	**4**	**91.89**	**97.14**	**89.47**	**94.44**
a70 Ab-1	140	127	13	11	90.71	92.03	84.11	91,37
**a70 Ab-2**	**140**	**137**	**3**	**2**	**97.86**	**98.56**	**96.48**	**98.21**
**a72 Ab-1**	**166**	**166**	**0**	**0**	**100**	**100**	**100**	**100**
a72 Ab-2	166	166	0	0	100	100	100	100
a72 Ab-3	166	164	2	1	98.80	99.39	98.20	99.09
a72 Ab-4	166	161	5	3	96.99	98.17	95.27	97.58
**Total**	9,103	8,876	227	123	97.51	98.63	96.21	98.07
**Best channel**	3,627	3,552	75	32	97.93	99.11	97.08	98.52

**Fig 9:**
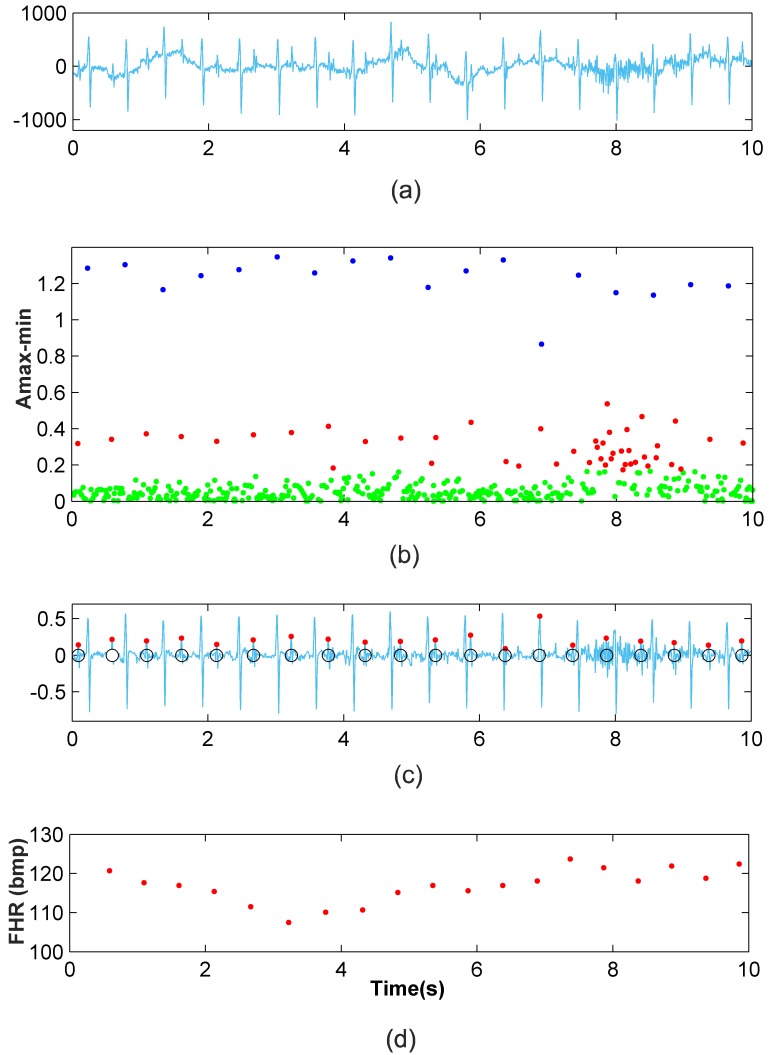
Application of the new method to the a03 Ab-4 recording. (a) 10-second recording (b) Clustering classification (c) Fetal RS detection after classification improvement and FP and FN correction (d) Ten-second FHR monitoring.

**Fig 10:**
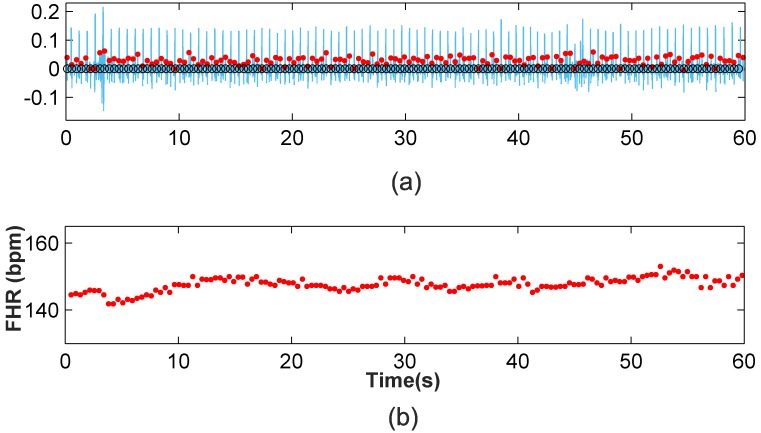
Application of the new method to the a49 Ab-2 recording. (a) Fetal RS detection after classification improvement and FP and FN correction (b) One-minute FHR monitoring for a49 Ab-2 recording.

Fig 9a depicts a segment of the a03 Ab-4 signal, in which it can be seen that the signal is contaminated by wandering, and the noise virtually masks the fetal QRS complexes in the interval from 7s to 9s. Fig 9c shows the denoised signal, the detected fetal R-peaks (red dots) and the clinical annotations (black circles), while Fig 9d presents the FHR monitoring of this signal. It is shown that apart from the wandering and noise, the FHR extraction method has a high accuracy. In the 7-9s interval, the classification improvement step and FP and FN correction step significantly improve the results from the clustering classification. This verifies the performance of our method in noisy scenarios of typical recordings. In addition, the 1-minute FHR monitoring of the signal a49 Ab-2 is presented in Fig 10b, where high efficiency can be also noted (100%). Moreover, the natural fluctuations in the FHR can be also noted. These results validate the proposal and provide evidence of its capabilities.

The presented proposal is thus able to perform robust fetal QRS detections for a total of 55 minutes for the *Abdominal and Direct Fetal Electrocardiogram Database* and 64 min for the *Challenge 2013 Training Set A*. The obtained results can be compared to those obtained from some recent single-channel fetal ECG extraction methods, including sequential total variation denoising (STVD), extended Kalman filter (EKF), template subtraction principle component analysis (TSPCA) and total variation denoising (TVD) combined to TSPCA (TVD+TSPCA) as proposed in [35], template substraction principle component analysis (TSpca), least mean square (LMS), recursive least square (RLS) and state neural network (ESNa), as proposed in [34], template adaption (TA) and extended Kalman smoother (EKS), as proposed in [36], extended Kalman smoother (EKS) combined to differential evolution (DE) and adaptive neuro fuzzy inference system (ANFIS) (EKS+DE+ANFIS) and EKF combined to DE and ANFIS (EKF+DE+ANFIS) as proposed in [8], singular value decomposition (SDV) combined to smoothd windows (SW) (SDV+SW) as proposed in [9], and complete ensemble empirical mode decomposition with adaptive noise (CEEMDAN) as proposed in [13]. Results are also compared to the threshold-based method (THR) proposed in [19]. This comparison is summarized in Table 3, where the recordings used for each method are indicated. Table 3 shows that the results obtained by the proposed FHR monitoring model exhibit improved success rates compared to those including a significant number and duration of recordings, such as STDV, EKF, TVD+TSPCA, TSPCA, TSLpca, LMS, RLS, ESNa, EKS+DE+ANFIS and EKF+DE+ANFIS methods. This good performance of the presented work compared to these approaches reaffirms its validity. The SDV+SW and CEEMDAN methods show a very high efficiency, but it must be noted that the results for the SDV+SW method are based on only 3 recordings, while results for the CEEMDAN method include only a few recordings of limited length, 30 and 40 seconds. It must be also noted that for our method, the testing database results are similar to those obtained for the training database, which shows the strength of our proposal.

**Table 3:** Comparison of the results of different FHR extraction methods.

Method	Dataset	*Se*(%)	*PPV*(%)	*F*_1_(%)
STVD [35]	68 1-minute recordings	90.50	89.4	89.90
EKF [35]	68 1-minute recordings	82.20	80.10	81.10
TVD+TSPCA [35]	68 1-minute recordings	87.70	87.20	87.40
TSPCA [35]	68 1-minute recordings	87.20	86.10	86.70
TSLpca [34]	14 1-minute recordings	94.70	96.00	95.04
	11 5-minute recordings	89.90	88.80	89.30
LMS [34]	14 1-minute recordings	95.80	95.00	95.40
	11 5-minute recordings	89.30	86.50	87.90
RLS [34]	14 1-minute recordings	96.20	95.60	95.90
	11 5-minute recordings	89.70	86.80	88.20
ESNa [34]	14 1-minute recordings	97.20	97.30	97.20
	11 5-minute recordings	91.40	88.90	90.20
TA [36]	75 1-minute recordings	97.40	97.20	97.30
	24 20-minute recordings	85.80	85.00	85.40
EKS [36]	75 1-minute recordings	93.10	92.80	93.00
	24 20-minute recordings	86.80	85.90	86.30
EKS+DE+ANFIS [8]	54 1-minute recordings	94.21	96.05	95.12
	150 1-minute recordings	91.47	92.18	91.82
EKF+DE+ANFIS [8]	54 1-minute recordings	93.03	95.05	94.03
	150 1-minute recordings	88.88	91.80	90.32
SDV+SW [9]	1 5-minute recording	99.53	99.69	99.61
	1 5-minute recording	99.20	99.36	99.28
	1 1-minute 20-second recording	98.31	98.86	98.58
CEEMDAN [13]	5 30-second recordings	99.15	98.27	98.6
	4 30-second recordings	100	100	100
	5 40-second recordings	97.15	95.31	96.2
THR [19]	5 1-minute recordings	95.57	97.99	96.76
	5 5-minute recordings	98.28	98.19	98.24
	2 15-minute recordings	97.91	97.10	97.50
Clustering-based FHR monitoring	11 5-minute recordings	97.50	98.58	98.04
Signals selected by a cardiologist	64 1-minute recordings	97.51	98.63	98.07
Clustering-based FHR monitoring	5 5-minute recordings	98.40	98.86	98.63
Best performing channels	26 1-minute recordings	97.93	99.11	98.52

## Conclusions

In this paper, we propose a new method for fetal QRS extraction from single-channel abdominal ECGs of pregnant women. The proposal combines a previously developed technique for wavelet-based denoising with a new clustering-based procedure for the extraction of fetal QRS complexes, along with a final stage for FP and FN correction. This clustering procedure locates candidate RS-peaks as local maxima of the denoised signal followed by a minimum and extracts the amplitude and time distance features of these candidate peaks. These features are used for the classification of candidate peaks into three clusters: maternal RS-peaks, fetal RS-peaks and other waves. A classification improvement step based on the amplitude distribution is also applied to increase efficiency. Parameters for the classification steps were optimized through the analysis of the *Abdominal and Direct Fetal ECG Database* from PhysioNet. Further testing and validation of the methodology has been carried out using real-life benchmark clinical recordings in the *Challenge 2013 Training Set A* dataset from PhysioNet. The obtained results illustrate how the proposed method achieves high accuracy in fetal QRS extraction for both training and testing databases, comprising a total of 119 minutes of abdominal data. It must be noted the fact that, while method parameters where tuned for the training recordings, results for the testing database are also highly accurate. This improves the applicability of the proposed method when compared to the threshold-based technique proposed in [19], while also enhancing the average accuracy in the detection of fetal QRS complexes. Comparisons to other existing FHR extraction methods also corroborate the high efficiency of the proposal. Finally, the good performance of the proposed method using a single parameter set over two different databases may allow the automated use of this method, which may also be proved to be useful for FHR monitoring in real-life clinical conditions with noisy abdominal ECG recordings.
